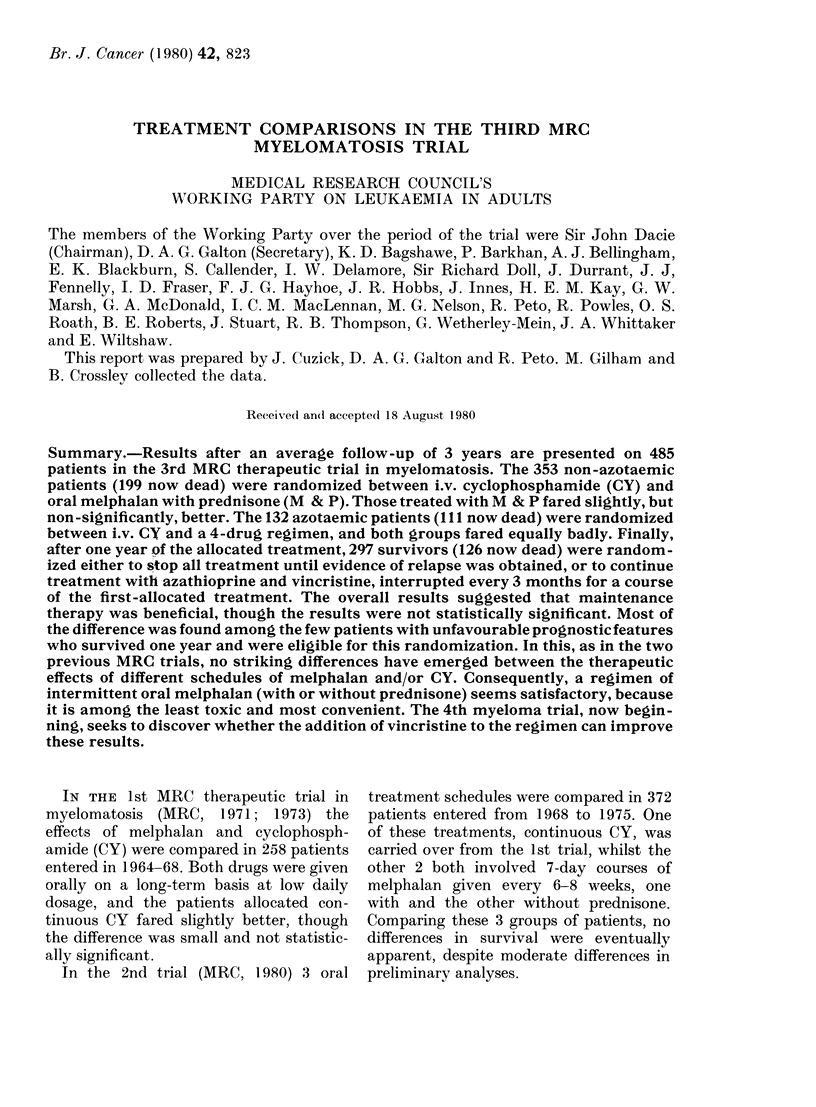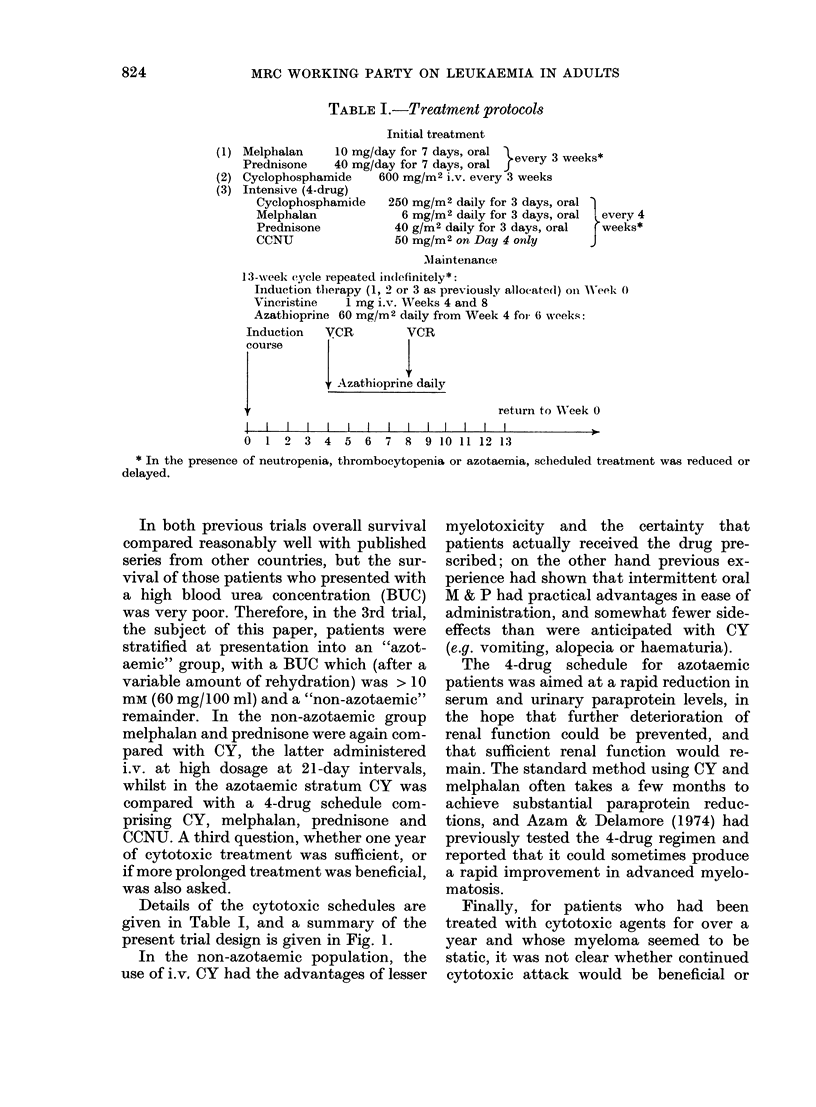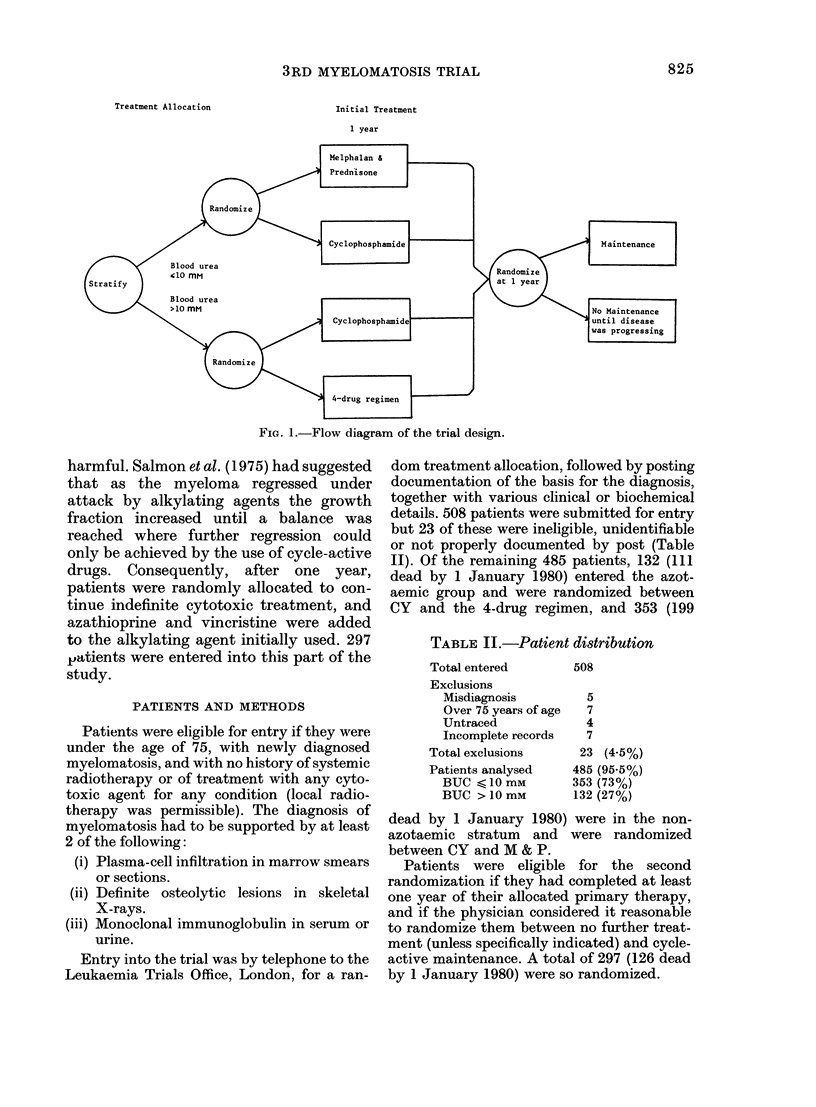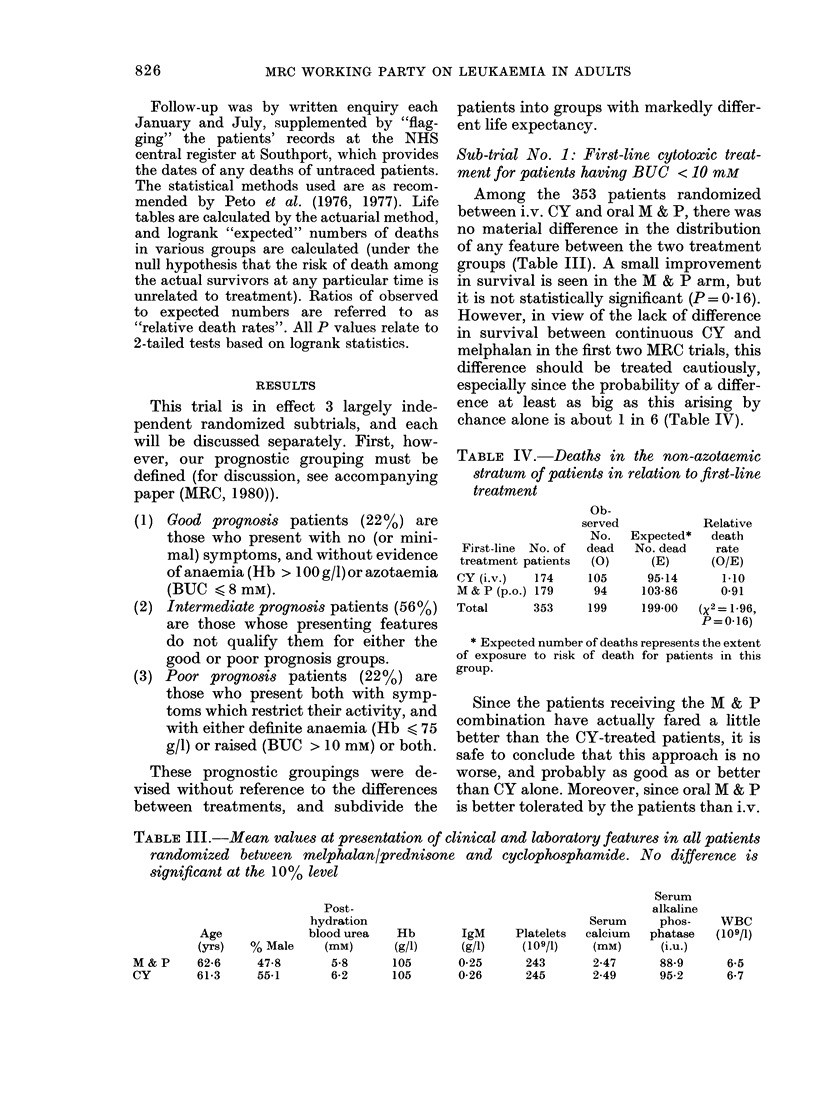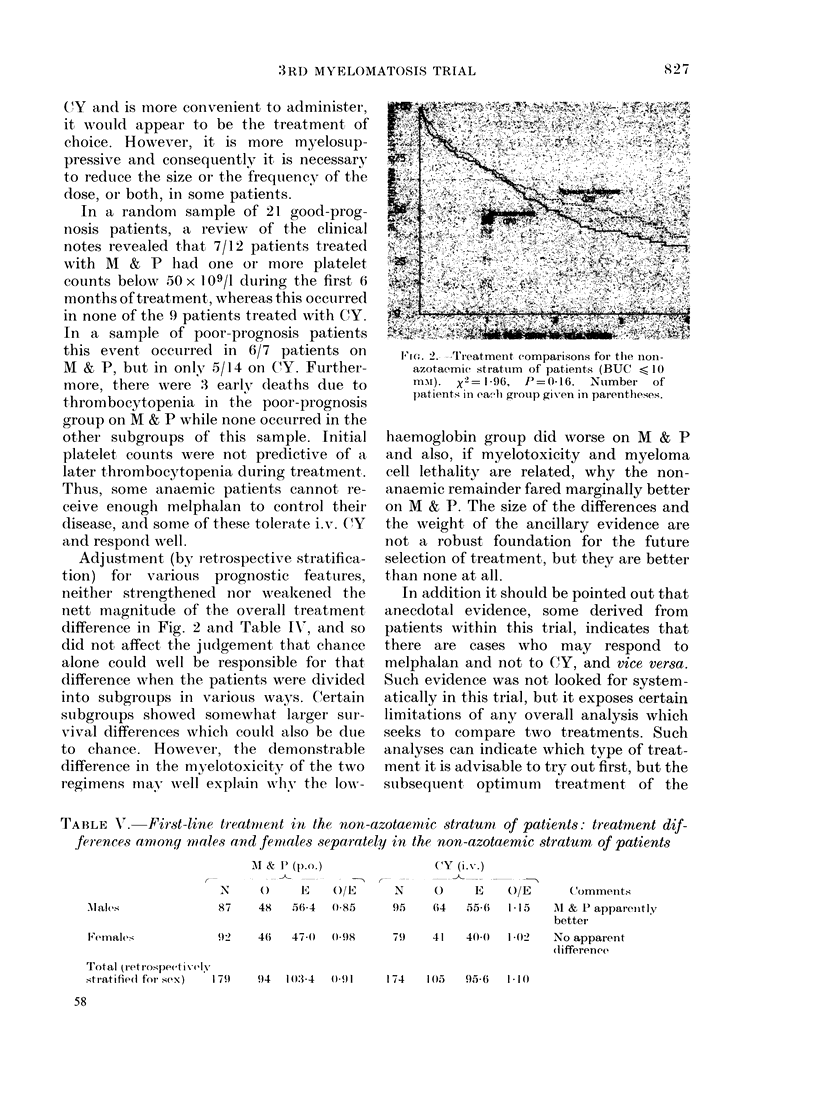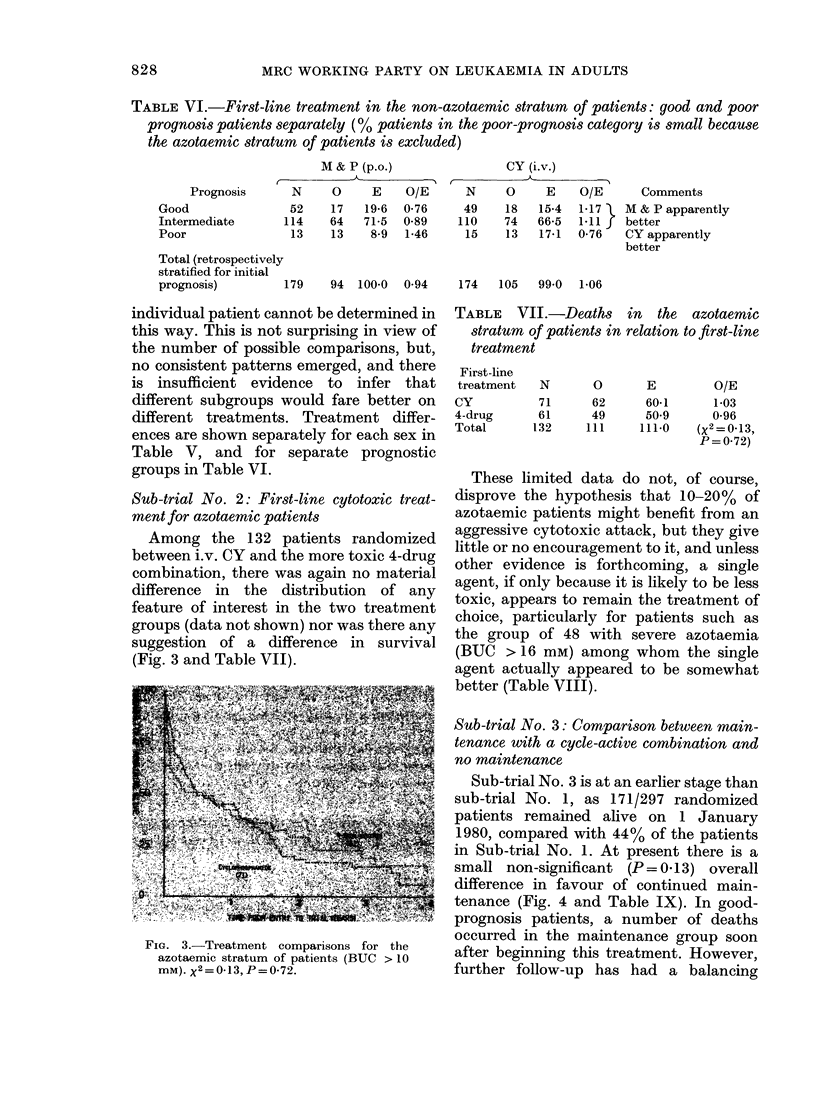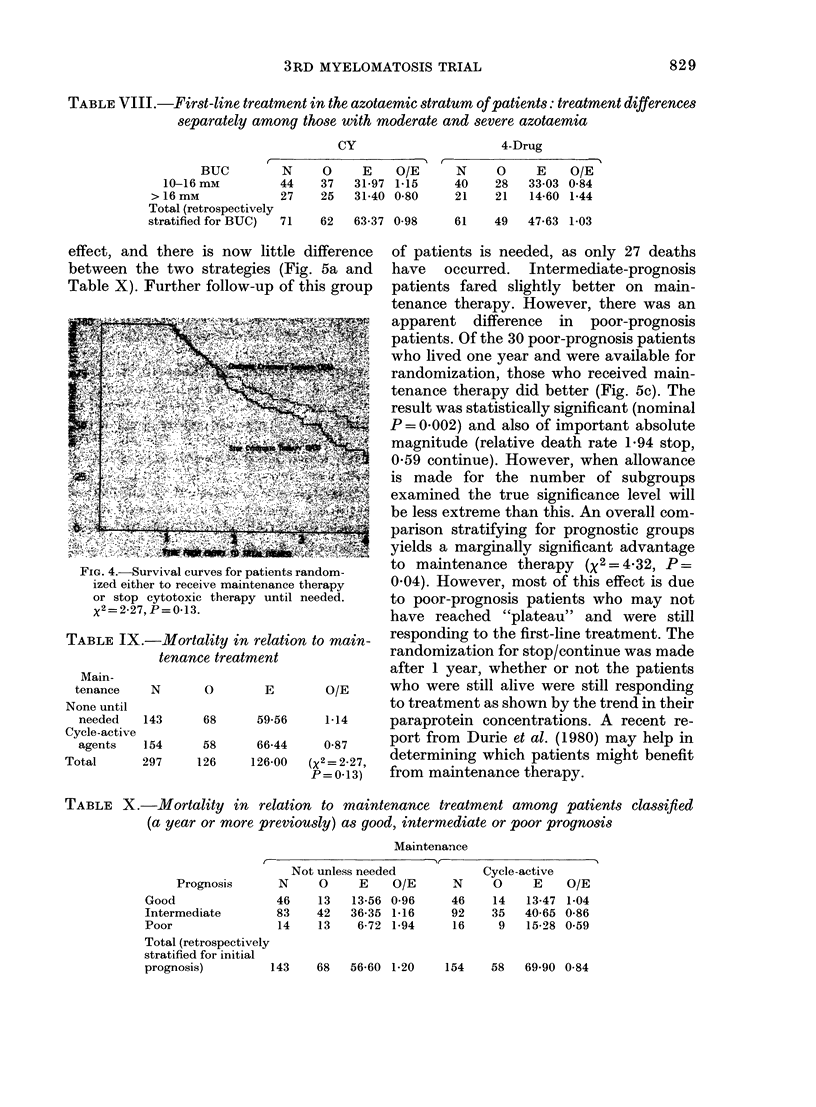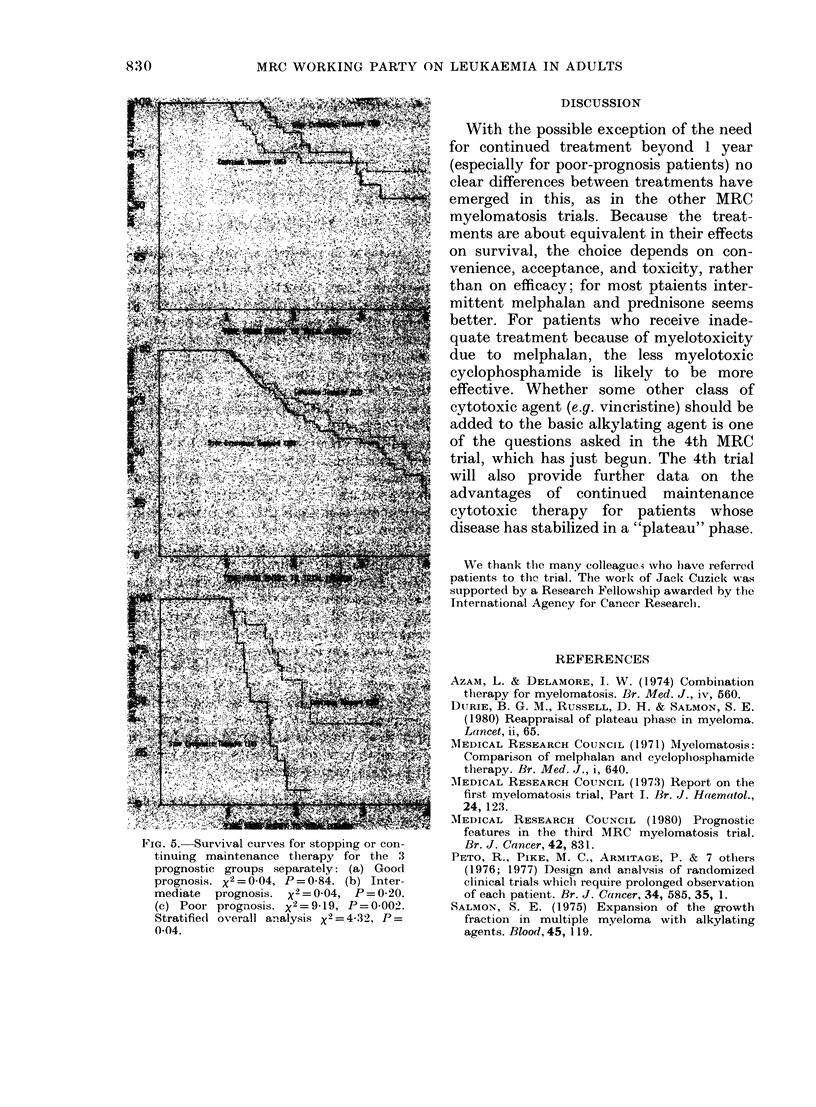# Treatment comparisons in the third MRC myelomatosis trial. Medical Research Council's Working Party on Leukaemia in Adults.

**DOI:** 10.1038/bjc.1980.329

**Published:** 1980-12

**Authors:** 

## Abstract

Results after an average follow-up of 3 years are presented on 485 patients in the 3rd MRC therapeutic trial in myelomatosis. The 353 non-azotaemic patients (199 now dead) were randomized between i.v. cyclophosphamide (CY) and oral melphalan with prednisone (M & P). THose treated with M & P fared slightly, but non-significantly, better. The 132 azotaemic patients (111 now dead) were randomized between i.v. CY and a 4-drug regimen, and both groups fared equally badly. Finally, after one year of the allocated treatment, 297 survivors (126 now dead) were randomized either to stop all treatment until evidence of relapse was obtained, or to continue treatment with azathioprine and vincristine, interrupted every 3 months for a course of the first-allocated treatment. The overall results suggested that maintenance therapy was beneficial, though the results were not statistically significant. Most of the difference was found among the few patients with unfavourable prognostic features who survived one year and were eligible for this randomization. In this, as in the two previous MRC trials, no striking differences have emerged between the therapeutic effects of different schedules of melphalan and/or CY. Consequently, a regimen of intermittent oral melphalan (with or without prednisone) seems satisfactory, because it is among the least toxic and most convenient. The 4th myeloma trial, now beginning, seeks to discover whether the addition of vincristine to the regimen can improve these results.


Br. J. Cancer (1980) 42, 823

### TREATMENT COMPARISONS IN THE THIRD MRC MYELOMATOSIS TRIAL

MEDICAL RESEARCH COUNCIL'S
WORKING PARTY ON LEUKAEMIA IN ADULTS

The members of the Working Party over the period of the trial were Sir John Dacie (Chairman), D. A. G. Galton (Secretary), K. D. Bagshawe, P. Barkhan, A. J. Bellingham, E. K. Blackburn, S. Callender, I. W. Delamore, Sir Richard Doll, J. Durrant, J. J, Fennelly, I. D. Fraser, F. J. G. Hayhoe, J. R. Hobbs, J. Innes, H. E. M. Kay, G. W. Marsh, G. A. McDonald, I. C. M. MacLennan, M. G. Nelson, R. Peto, R. Powles, O. S. Roath, B. E. Roberts, J. Stuart, R. B. Thompson, G. Wetherley-Mein, J. A. Whittaker and E. Wiltshaw.

This report was prepared by J. Cuzick, D. A. G. Galton and R. Peto. M. Gilham and B. Crossley collected the data.

Received and accepted 18 August 1980

**Summary.—Results after an average follow-up of 3 years are presented on 485 patients in the 3rd MRC therapeutic trial in myelomatosis. The 353 non-azotaemic patients (199 now dead) were randomized between i.v. cyclophosphamide (CY) and oral melphalan with prednisone (M & P). Those treated with M & P fared slightly, but non-significantly, better. The 132 azotaemic patients (111 now dead) were randomized between i.v. CY and a 4-drug regimen, and both groups fared equally badly. Finally, after one year of the allocated treatment, 297 survivors (126 now dead) were randomized either to stop all treatment until evidence of relapse was obtained, or to continue treatment with azathioprine and vincristine, interrupted every 3 months for a course of the first-allocated treatment. The overall results suggested that maintenance therapy was beneficial, though the results were not statistically significant. Most of the difference was found among the few patients with unfavourable prognostic features who survived one year and were eligible for this randomization. In this, as in the two previous MRC trials, no striking differences have emerged between the therapeutic effects of different schedules of melphalan and/or CY. Consequently, a regimen of intermittent oral melphalan (with or without prednisone) seems satisfactory, because it is among the least toxic and most convenient. The 4th myeloma trial, now beginning, seeks to discover whether the addition of vincristine to the regimen can improve these results.**

In the 1st MRC therapeutic trial in myelomatosis (MRC, 1971; 1973) the effects of melphalan and cyclophosphamide (CY) were compared in 258 patients entered in 1964–68. Both drugs were given orally on a long-term basis at low daily dosage, and the patients allocated continuous CY fared slightly better, though the difference was small and not statistically significant.

In the 2nd trial (MRC, 1980) 3 oral treatment schedules were compared in 372 patients entered from 1968 to 1975. One of these treatments, continuous CY, was carried over from the 1st trial, whilst the other 2 both involved 7-day courses of melphalan given every 6–8 weeks, one with and the other without prednisone. Comparing these 3 groups of patients, no differences in survival were eventually apparent, despite moderate differences in preliminary analyses.

TABLE I.—*Treatment protocols*

Initial treatment

| | | | |
|---|---|---|---|
| (1) | Melphalan | 10 mg/day for 7 days, oral | every 3 weeks* |
| | Prednisone | 40 mg/day for 7 days, oral | |
| (2) | Cyclophosphamide | 600 mg/m$^2$ i.v. every 3 weeks | |
| (3) | Intensive (4-drug) | | |
| | Cyclophosphamide | 250 mg/m$^2$ daily for 3 days, oral | every 4 weeks* |
| | Melphalan | 6 mg/m$^2$ daily for 3 days, oral | |
| | Prednisone | 40 g/m$^2$ daily for 3 days, oral | |
| | CCNU | 50 mg/m$^2$ *on Day 4 only* | |

Maintenance

13-week cycle repeated indefinitely*:
Induction therapy (1, 2 or 3 as previously allocated) on Week 0
Vincristine 1 mg i.v. Weeks 4 and 8
Azathioprine 60 mg/m$^2$ daily from Week 4 for 6 weeks:

* In the presence of neutropenia, thrombocytopenia or azotaemia, scheduled treatment was reduced or delayed.

In both previous trials overall survival compared reasonably well with published series from other countries, but the survival of those patients who presented with a high blood urea concentration (BUC) was very poor. Therefore, in the 3rd trial, the subject of this paper, patients were stratified at presentation into an "azotaemic" group, with a BUC which (after a variable amount of rehydration) was > 10 mM (60 mg/100 ml) and a "non-azotaemic" remainder. In the non-azotaemic group melphalan and prednisone were again compared with CY, the latter administered i.v. at high dosage at 21-day intervals, whilst in the azotaemic stratum CY was compared with a 4-drug schedule comprising CY, melphalan, prednisone and CCNU. A third question, whether one year of cytotoxic treatment was sufficient, or if more prolonged treatment was beneficial, was also asked.

Details of the cytotoxic schedules are given in Table I, and a summary of the present trial design is given in Fig. 1.

In the non-azotaemic population, the use of i.v. CY had the advantages of lesser myelotoxicity and the certainty that patients actually received the drug prescribed; on the other hand previous experience had shown that intermittent oral M & P had practical advantages in ease of administration, and somewhat fewer side-effects than were anticipated with CY (*e.g.* vomiting, alopecia or haematuria).

The 4-drug schedule for azotaemic patients was aimed at a rapid reduction in serum and urinary paraprotein levels, in the hope that further deterioration of renal function could be prevented, and that sufficient renal function would remain. The standard method using CY and melphalan often takes a few months to achieve substantial paraprotein reductions, and Azam & Delamore (1974) had previously tested the 4-drug regimen and reported that it could sometimes produce a rapid improvement in advanced myelomatosis.

Finally, for patients who had been treated with cytotoxic agents for over a year and whose myeloma seemed to be static, it was not clear whether continued cytotoxic attack would be beneficial or

FIG. 1.—Flow diagram of the trial design.

harmful. Salmon *et al.* (1975) had suggested that as the myeloma regressed under attack by alkylating agents the growth fraction increased until a balance was reached where further regression could only be achieved by the use of cycle-active drugs. Consequently, after one year, patients were randomly allocated to continue indefinite cytotoxic treatment, and azathioprine and vincristine were added to the alkylating agent initially used. 297 patients were entered into this part of the study.

### PATIENTS AND METHODS

Patients were eligible for entry if they were under the age of 75, with newly diagnosed myelomatosis, and with no history of systemic radiotherapy or of treatment with any cytotoxic agent for any condition (local radiotherapy was permissible). The diagnosis of myelomatosis had to be supported by at least 2 of the following:

(i) Plasma-cell infiltration in marrow smears or sections.
(ii) Definite osteolytic lesions in skeletal X-rays.
(iii) Monoclonal immunoglobulin in serum or urine.

Entry into the trial was by telephone to the Leukaemia Trials Office, London, for a random treatment allocation, followed by posting documentation of the basis for the diagnosis, together with various clinical or biochemical details. 508 patients were submitted for entry but 23 of these were ineligible, unidentifiable or not properly documented by post (Table II). Of the remaining 485 patients, 132 (111 dead by 1 January 1980) entered the azotaemic group and were randomized between CY and the 4-drug regimen, and 353 (199 dead by 1 January 1980) were in the non-azotaemic stratum and were randomized between CY and M & P.

TABLE II.—*Patient distribution*

| | |
|---|---|
| Total entered | 508 |
| Exclusions | |
| Misdiagnosis | 5 |
| Over 75 years of age | 7 |
| Untraced | 4 |
| Incomplete records | 7 |
| Total exclusions | 23 (4·5%) |
| Patients analysed | 485 (95·5%) |
| BUC ≤ 10 mM | 353 (73%) |
| BUC > 10 mM | 132 (27%) |

Patients were eligible for the second randomization if they had completed at least one year of their allocated primary therapy, and if the physician considered it reasonable to randomize them between no further treatment (unless specifically indicated) and cycle-active maintenance. A total of 297 (126 dead by 1 January 1980) were so randomized.

Follow-up was by written enquiry each January and July, supplemented by "flagging" the patients' records at the NHS central register at Southport, which provides the dates of any deaths of untraced patients. The statistical methods used are as recommended by Peto *et al.* (1976, 1977). Life tables are calculated by the actuarial method, and logrank "expected" numbers of deaths in various groups are calculated (under the null hypothesis that the risk of death among the actual survivors at any particular time is unrelated to treatment). Ratios of observed to expected numbers are referred to as "relative death rates". All $P$ values relate to 2-tailed tests based on logrank statistics.

### RESULTS

This trial is in effect 3 largely independent randomized subtrials, and each will be discussed separately. First, however, our prognostic grouping must be defined (for discussion, see accompanying paper (MRC, 1980)).

(1) *Good prognosis* patients (22%) are those who present with no (or minimal) symptoms, and without evidence of anaemia (Hb > 100 g/l) or azotaemia (BUC ⩽ 8 mM).

(2) *Intermediate prognosis* patients (56%) are those whose presenting features do not qualify them for either the good or poor prognosis groups.

(3) *Poor prognosis* patients (22%) are those who present both with symptoms which restrict their activity, and with either definite anaemia (Hb ⩽ 75 g/l) or raised (BUC > 10 mM) or both.

These prognostic groupings were devised without reference to the differences between treatments, and subdivide the patients into groups with markedly different life expectancy.

*Sub-trial No. 1: First-line cytotoxic treatment for patients having BUC < 10 mM*

Among the 353 patients randomized between i.v. CY and oral M & P, there was no material difference in the distribution of any feature between the two treatment groups (Table III). A small improvement in survival is seen in the M & P arm, but it is not statistically significant ($P = 0{\cdot}16$). However, in view of the lack of difference in survival between continuous CY and melphalan in the first two MRC trials, this difference should be treated cautiously, especially since the probability of a difference at least as big as this arising by chance alone is about 1 in 6 (Table IV).

TABLE IV.—*Deaths in the non-azotaemic stratum of patients in relation to first-line treatment*

| First-line treatment | No. of patients | Observed No. dead (O) | Expected* No. dead (E) | Relative death rate (O/E) |
|---|---|---|---|---|
| CY (i.v.) | 174 | 105 | 95·14 | 1·10 |
| M & P (p.o.) | 179 | 94 | 103·86 | 0·91 |
| Total | 353 | 199 | 199·00 | ($\chi^2 = 1{\cdot}96$, $P = 0{\cdot}16$) |

* Expected number of deaths represents the extent of exposure to risk of death for patients in this group.

Since the patients receiving the M & P combination have actually fared a little better than the CY-treated patients, it is safe to conclude that this approach is no worse, and probably as good as or better than CY alone. Moreover, since oral M & P is better tolerated by the patients than i.v.

TABLE III.—*Mean values at presentation of clinical and laboratory features in all patients randomized between melphalan/prednisone and cyclophosphamide. No difference is significant at the* 10% *level*

| | Age (yrs) | % Male | Post-hydration blood urea (mM) | Hb (g/l) | IgM (g/l) | Platelets ($10^9$/l) | Serum calcium (mM) | Serum alkaline phosphatase (i.u.) | WBC ($10^9$/l) |
|---|---|---|---|---|---|---|---|---|---|
| M & P | 62·6 | 47·8 | 5·8 | 105 | 0·25 | 243 | 2·47 | 88·9 | 6·5 |
| CY | 61·3 | 55·1 | 6·2 | 105 | 0·26 | 245 | 2·49 | 95·2 | 6·7 |

FIG. 2.—Treatment comparisons for the non-azotaemic stratum of patients (BUC $\leqslant 10$ mM). $\chi^2 = 1 \cdot 96$, $P = 0 \cdot 16$. Number of patients in each group given in parentheses.

CY and is more convenient to administer, it would appear to be the treatment of choice. However, it is more myelosuppressive and consequently it is necessary to reduce the size or the frequency of the dose, or both, in some patients.

In a random sample of 21 good-prognosis patients, a review of the clinical notes revealed that 7/12 patients treated with M & P had one or more platelet counts below $50 \times 10^9$/l during the first 6 months of treatment, whereas this occurred in none of the 9 patients treated with CY. In a sample of poor-prognosis patients this event occurred in 6/7 patients on M & P, but in only 5/14 on CY. Furthermore, there were 3 early deaths due to thrombocytopenia in the poor-prognosis group on M & P while none occurred in the other subgroups of this sample. Initial platelet counts were not predictive of a later thrombocytopenia during treatment. Thus, some anaemic patients cannot receive enough melphalan to control their disease, and some of these tolerate i.v. CY and respond well.

Adjustment (by retrospective stratification) for various prognostic features, neither strengthened nor weakened the nett magnitude of the overall treatment difference in Fig. 2 and Table IV, and so did not affect the judgement that chance alone could well be responsible for that difference when the patients were divided into subgroups in various ways. Certain subgroups showed somewhat larger survival differences which could also be due to chance. However, the demonstrable difference in the myelotoxicity of the two regimens may well explain why the low-haemoglobin group did worse on M & P and also, if myelotoxicity and myeloma cell lethality are related, why the non-anaemic remainder fared marginally better on M & P. The size of the differences and the weight of the ancillary evidence are not a robust foundation for the future selection of treatment, but they are better than none at all.

In addition it should be pointed out that anecdotal evidence, some derived from patients within this trial, indicates that there are cases who may respond to melphalan and not to CY, and *vice versa*. Such evidence was not looked for systematically in this trial, but it exposes certain limitations of any overall analysis which seeks to compare two treatments. Such analyses can indicate which type of treatment it is advisable to try out first, but the subsequent optimum treatment of the

TABLE V.—*First-line treatment in the non-azotaemic stratum of patients: treatment differences among males and females separately in the non-azotaemic stratum of patients*

| | M & P (p.o.) | | | | CY (i.v.) | | | | |
|---|---|---|---|---|---|---|---|---|---|
| | N | O | E | O/E | N | O | E | O/E | Comments |
| Males | 87 | 48 | 56·4 | 0·85 | 95 | 64 | 55·6 | 1·15 | M & P apparently better |
| Females | 92 | 46 | 47·0 | 0·98 | 79 | 41 | 40·0 | 1·02 | No apparent difference |
| Total (retrospectively stratified for sex) | 179 | 94 | 103·4 | 0·91 | 174 | 105 | 95·6 | 1·10 | |

TABLE VI.—*First-line treatment in the non-azotaemic stratum of patients: good and poor prognosis patients separately (% patients in the poor-prognosis category is small because the azotaemic stratum of patients is excluded)*

| | M & P (p.o.) | | | | CY (i.v.) | | | | |
|---|---|---|---|---|---|---|---|---|---|
| Prognosis | N | O | E | O/E | N | O | E | O/E | Comments |
| Good | 52 | 17 | 19·6 | 0·76 | 49 | 18 | 15·4 | 1·17 | M & P apparently better (Good and Intermediate) |
| Intermediate | 114 | 64 | 71·5 | 0·89 | 110 | 74 | 66·5 | 1·11 | |
| Poor | 13 | 13 | 8·9 | 1·46 | 15 | 13 | 17·1 | 0·76 | CY apparently better |
| Total (retrospectively stratified for initial prognosis) | 179 | 94 | 100·0 | 0·94 | 174 | 105 | 99·0 | 1·06 | |

individual patient cannot be determined in this way. This is not surprising in view of the number of possible comparisons, but, no consistent patterns emerged, and there is insufficient evidence to infer that different subgroups would fare better on different treatments. Treatment differences are shown separately for each sex in Table V, and for separate prognostic groups in Table VI.

*Sub-trial No. 2: First-line cytotoxic treatment for azotaemic patients*

Among the 132 patients randomized between i.v. CY and the more toxic 4-drug combination, there was again no material difference in the distribution of any feature of interest in the two treatment groups (data not shown) nor was there any suggestion of a difference in survival (Fig. 3 and Table VII).

FIG. 3.—Treatment comparisons for the azotaemic stratum of patients (BUC > 10 mM). $\chi^2=0{\cdot}13$, $P=0{\cdot}72$.

TABLE VII.—*Deaths in the azotaemic stratum of patients in relation to first-line treatment*

| First-line treatment | N | O | E | O/E |
|---|---|---|---|---|
| CY | 71 | 62 | 60·1 | 1·03 |
| 4-drug | 61 | 49 | 50·9 | 0·96 |
| Total | 132 | 111 | 111·0 | ($\chi^2=0{\cdot}13$, $P=0{\cdot}72$) |

These limited data do not, of course, disprove the hypothesis that 10–20% of azotaemic patients might benefit from an aggressive cytotoxic attack, but they give little or no encouragement to it, and unless other evidence is forthcoming, a single agent, if only because it is likely to be less toxic, appears to remain the treatment of choice, particularly for patients such as the group of 48 with severe azotaemia (BUC > 16 mM) among whom the single agent actually appeared to be somewhat better (Table VIII).

*Sub-trial No. 3: Comparison between maintenance with a cycle-active combination and no maintenance*

Sub-trial No. 3 is at an earlier stage than sub-trial No. 1, as 171/297 randomized patients remained alive on 1 January 1980, compared with 44% of the patients in Sub-trial No. 1. At present there is a small non-significant ($P=0{\cdot}13$) overall difference in favour of continued maintenance (Fig. 4 and Table IX). In good-prognosis patients, a number of deaths occurred in the maintenance group soon after beginning this treatment. However, further follow-up has had a balancing

TABLE VIII.—*First-line treatment in the azotaemic stratum of patients: treatment differences separately among those with moderate and severe azotaemia*

| | CY | | | | 4-Drug | | | |
|---|---|---|---|---|---|---|---|---|
| BUC | N | O | E | O/E | N | O | E | O/E |
| 10–16 mM | 44 | 37 | 31·97 | 1·15 | 40 | 28 | 33·03 | 0·84 |
| > 16 mM | 27 | 25 | 31·40 | 0·80 | 21 | 21 | 14·60 | 1·44 |
| Total (retrospectively stratified for BUC) | 71 | 62 | 63·37 | 0·98 | 61 | 49 | 47·63 | 1·03 |

effect, and there is now little difference between the two strategies (Fig. 5a and Table X). Further follow-up of this group of patients is needed, as only 27 deaths have occurred. Intermediate-prognosis patients fared slightly better on maintenance therapy. However, there was an apparent difference in poor-prognosis patients. Of the 30 poor-prognosis patients who lived one year and were available for randomization, those who received maintenance therapy did better (Fig. 5c). The result was statistically significant (nominal $P = 0{\cdot}002$) and also of important absolute magnitude (relative death rate 1·94 stop, 0·59 continue). However, when allowance is made for the number of subgroups examined the true significance level will be less extreme than this. An overall comparison stratifying for prognostic groups yields a marginally significant advantage to maintenance therapy ($\chi^2 = 4{\cdot}32$, $P = 0{\cdot}04$). However, most of this effect is due to poor-prognosis patients who may not have reached "plateau" and were still responding to the first-line treatment. The randomization for stop/continue was made after 1 year, whether or not the patients who were still alive were still responding to treatment as shown by the trend in their paraprotein concentrations. A recent report from Durie *et al.* (1980) may help in determining which patients might benefit from maintenance therapy.

FIG. 4.—Survival curves for patients randomized either to receive maintenance therapy or stop cytotoxic therapy until needed. $\chi^2 = 2{\cdot}27$, $P = 0{\cdot}13$.

TABLE IX.—*Mortality in relation to maintenance treatment*

| Maintenance | N | O | E | O/E |
|---|---|---|---|---|
| None until needed | 143 | 68 | 59·56 | 1·14 |
| Cycle-active agents | 154 | 58 | 66·44 | 0·87 |
| Total | 297 | 126 | 126·00 | ($\chi^2 = 2{\cdot}27$, $P = 0{\cdot}13$) |

TABLE X.—*Mortality in relation to maintenance treatment among patients classified (a year or more previously) as good, intermediate or poor prognosis*

| | Maintenance | | | | | | | |
|---|---|---|---|---|---|---|---|---|
| | Not unless needed | | | | Cycle-active | | | |
| Prognosis | N | O | E | O/E | N | O | E | O/E |
| Good | 46 | 13 | 13·56 | 0·96 | 46 | 14 | 13·47 | 1·04 |
| Intermediate | 83 | 42 | 36·35 | 1·16 | 92 | 35 | 40·65 | 0·86 |
| Poor | 14 | 13 | 6·72 | 1·94 | 16 | 9 | 15·28 | 0·59 |
| Total (retrospectively stratified for initial prognosis) | 143 | 68 | 56·60 | 1·20 | 154 | 58 | 69·90 | 0·84 |

FIG. 5.—Survival curves for stopping or continuing maintenance therapy for the 3 prognostic groups separately: (a) Good prognosis. $\chi^2=0\cdot04$, $P=0\cdot84$. (b) Intermediate prognosis. $\chi^2=0\cdot04$, $P=0\cdot20$. (c) Poor prognosis. $\chi^2=9\cdot19$, $P=0\cdot002$. Stratified overall analysis $\chi^2=4\cdot32$, $P=0\cdot04$.

### DISCUSSION

With the possible exception of the need for continued treatment beyond 1 year (especially for poor-prognosis patients) no clear differences between treatments have emerged in this, as in the other MRC myelomatosis trials. Because the treatments are about equivalent in their effects on survival, the choice depends on convenience, acceptance, and toxicity, rather than on efficacy; for most ptaients intermittent melphalan and prednisone seems better. For patients who receive inadequate treatment because of myelotoxicity due to melphalan, the less myelotoxic cyclophosphamide is likely to be more effective. Whether some other class of cytotoxic agent (*e.g.* vincristine) should be added to the basic alkylating agent is one of the questions asked in the 4th MRC trial, which has just begun. The 4th trial will also provide further data on the advantages of continued maintenance cytotoxic therapy for patients whose disease has stabilized in a "plateau" phase.

We thank the many colleagues who have referred patients to the trial. The work of Jack Cuzick was supported by a Research Fellowship awarded by the International Agency for Cancer Research.

### REFERENCES

AZAM, L. & DELAMORE, I. W. (1974) Combination therapy for myelomatosis. *Br. Med. J.*, iv, 560.

DURIE, B. G. M., RUSSELL, D. H. & SALMON, S. E. (1980) Reappraisal of plateau phase in myeloma. *Lancet*, ii, 65.

MEDICAL RESEARCH COUNCIL (1971) Myelomatosis: Comparison of melphalan and cyclophosphamide therapy. *Br. Med. J.*, i, 640.

MEDICAL RESEARCH COUNCIL (1973) Report on the first myelomatosis trial, Part I. *Br. J. Haematol.*, **24,** 123.

MEDICAL RESEARCH COUNCIL (1980) Prognostic features in the third MRC myelomatosis trial. *Br. J. Cancer*, **42,** 831.

PETO, R., PIKE, M. C., ARMITAGE, P. & 7 others (1976; 1977) Design and analysis of randomized clinical trials which require prolonged observation of each patient. *Br. J. Cancer*, **34,** 585, **35,** 1.

SALMON, S. E. (1975) Expansion of the growth fraction in multiple myeloma with alkylating agents. *Blood*, **45,** 119.